# Editor’s view: What makes science successful?

**DOI:** 10.7189/jogh.15.01005

**Published:** 2025-07-21

**Authors:** Igor Rudan

**Affiliations:** 1 Centre for Global Health, Usher Institute, The University of Edinburgh, UK; 2 Nuffield Department of Primary Care Health Sciences and Green Templeton College, Oxford University, UK

## Abstract

This editorial examines the factors contributing to the success of science, tracing its evolution from fundamental human curiosity to contemporary advancements propelled by technology, data, and artificial intelligence (AI). Beginning with the hypothesis-testing process, it highlights how imaginative individuals throughout history have offered explanations for the natural world, designed experiments, and amassed evidence to confirm or reject their ideas and theories, thus generating new knowledge and understanding of nature. Early humans formulated simple myths and legends as the first scientific hypotheses, partly to lessen their fear of the unknown. A more scientific turn appeared when rare explorer-scientists ventured beyond their ancestral homes, gathered empirical information using their limited senses, made choices based on observations, and sometimes relocated entire communities. Their efforts reflected the timeless elements of the scientific method: from generating a hypothesis to its experimental proof, broad validation and application of new knowledge. The paper then examines the characteristics of successful scientific disciplines. They attract many researchers who generate novel ideas and hypotheses, building an accelerating momentum of discovery. Further hallmarks of such fields are swift and fair peer validation and robust mechanisms for applying new knowledge to improve human well-being. By contrast, less successful fields will struggle with attracting talent, leading to slower progress, which could also be coupled with resistance to new ideas and obstacles to real-world translation of new knowledge. A central theme of the paper is the contribution of measurement and tools to science’s success. Modern instruments, from microscopes and telescopes to satellites and statistical tools, have extended our perception of nature, revealing realms far smaller and far larger than human senses can access. The paper also addresses the revolution of ‘hypothesis-free science’, driven by computers and big data. Rather than framing a single hypothesis, modern researchers gather enormous datasets and use algorithms to test large numbers of possible hypotheses simultaneously and systematically, free of human bias introduced through existing knowledge. Finally, the paper explores how AI could advance science to unprecedented successes: not just by improving human senses like a microscope does, providing additional ones like the Large Hadron Collider does, or extending human memory and computational capacity like computers do, but also by expanding human reasoning itself. Unlike previous tools, AI can synthesise human knowledge and generate hypotheses, design studies, explore patterns and write papers, thus becoming both a ‘philosopher 2.0’ and a ‘scientist 2.0’. Therefore, AI may transform science from a human-centred endeavour into collaborative effort that relies on hybrid intelligence. This unprecedented new frontier will require attention to questions of its explainability, bias, authorship, ethics, and accountability. In the future, science will remain successful by staying aligned with its fundamental mission: to improve the human condition through the expansion of knowledge and understanding of our world.

Throughout history, science has most often advanced through a process known as hypothesis testing. Hypotheses are scientific theories put forward by scientists, typically based on their existing knowledge, that offer explanations for something unknown in nature. Scientists who were sufficiently educated and imaginative would formulate intriguing hypotheses. They would then design experiments involving specific measurements, with the aim of generating new and useful information that might support or refute their hypotheses. Over time, as experimental data accumulated, certain hypotheses would gain broader acceptance, leading to the creation of new human knowledge and a deeper understanding of nature and the world we live in.

In this editorial, I explore the factors contributing to the success of science, tracing its evolution from fundamental human curiosity to contemporary advancements propelled by technology and data. I reflect on the critical importance of precise measurement for the development and progress of science. I also explain why, in addition to relying on our senses, we must depend on tools and instruments to expand human knowledge. In this context, I examine the factors that make a particular branch of science ‘successful’ and explain why its ultimate goal is the application of new knowledge in ways that improve the quality, safety, and duration of human life.

## SCIENTIFIC DISCOVERY AND THE CREATION OF NEW KNOWLEDGE

The earliest humans – our distant ancestors – did not lead refined or leisurely lives. Their daily routines closely resembled those of other wild creatures still roaming the wilderness today. Humans tried to adapt to their environment, but their understanding of it was extremely limited. All their knowledge about the world around them came through what they could see, hear, touch, taste, or smell. They used their brains to combine and interpret the information gathered through their senses, increasing their odds of survival from one day to the next. Memory of past experiences helped them make better decisions over time.

Yet, from today’s perspective, their condition was a harsh one. They did not live long. Many died as infants or young children due to exposure to microscopic life – bacteria or viruses – that caused fatal infections. Those who survived childhood soon had to start hunting daily just to feed themselves. That was a dangerous and exhausting task. Many fell victim to drowning, falling, bleeding, poisoning, or predation by larger beasts. At night, while hiding in their shelters, they had no knowledge of the molecular or hormonal processes involved in conception. Still, by following their instincts, they managed to have many children. Tragically, women often died during childbirth. In times of adverse climate, entire tribes would freeze to death or starve. Their lack of knowledge and poor understanding of the natural world made their lives very risky and their choices severely limited.

Even so, they likely wondered about their surroundings – about the deep, seemingly endless seas, the towering mountains, smoking volcanoes, rainfall and snow, desert sands, and the occasional fog. They were aware of the many kinds of plants that grew around them. They were also constantly on guard against many animals that could harm or kill them. They must have been puzzled by birds that could fly and fish that could live beneath the surface of rivers, lakes, and oceans. Some may have wondered where the water in rivers and lakes came from, what kinds of soil existed, what caused thunder and earthquakes, why sunlight gave way to night, and what the Moon and stars truly were. Based on what they observed in nature, the more imaginative among them would try to offer explanations for these mysterious phenomena. For this, they first needed to ‘invent’ language. Once the earliest words were adopted, their first explanations usually took the form of simple stories, which were passed down through generations and became legends. Many of these ancient stories were beautiful analogies, drawn from their shared experiences in nature, and were accepted in the absence of better explanations.

This lack of understanding of their context gave rise to deep fears of the unknown. Any explanation, no matter how accurate, was welcomed because it reduced this fear. These early legends were, in a sense, the first scientific hypotheses. Why? Because they were imaginative attempts, based on their existing knowledge and experience, to explain unknown natural events. Many cultures preserved such legends to explain the origin of the Sun and Moon, day and night, or rain and snow using familiar features from their own landscapes. One such example comes from the North American Indigenous tradition, titled ‘Wesakechak and the Origin of the Moon’ [1].

A long time ago, there was no Moon. There was only the Sun. The Creator had messengers who helped him in his work. One of these was the Caretaker of the Sun. He had two children, a boy and a girl. All three lived in the Sky World. They were very happy. The daughter looked after the camp. She kept it clean and tidy. When she shook the feather bedding, the feathers would fall to the Earth as snow. The son hunted and fished. When he hung his nets to dry, droplets fell to Earth as rain. The father would be away. All day he kept the great fire, burning on the Sun. He was very old. Soon he would leave his children, never to return. He said to them, “When I die, you must keep the fire burning, or else the people and animals on Earth will die.” One day when the fire was low on the Sun, the father came home tired. He said, “Children, my children, my children. I have to go. I will never return.” The children cried and mourned. They knew he would die. In the morning, it was time to start the Sun's fire. The children began to quarrel over who would do the task. “I will tend the fire, I am older,” said the sister. “No, I am the man, I will do it,” said the brother. They yelled thus to each other. The people on Earth began to worry, saying, “Why is the sun so late? It should be up by now!” Wesakechak went to the Sun to see what was the matter. When he arrived, the boy and his sister were still quarrelling. Wesakechak was angry. “The people and animals will perish,” he said to them. “It is up to you! You keep the fire burning,” he told the boy. “Your name from now on will be Pisim.” To the sister he said, “You, too, will work as hard as your brother. You will keep the fire in another place. You will work at night. You will be Tipiskawipisim, the Moon. The two of you did not get along. As a punishment, you will see each other once a year. For all time, you will see each other from across the sky.” And so it happened. Even now it is so.

Comforted by such stories and legends, our distant ancestors would come to terms with their fate and remain in their habitats. They would only consider moving elsewhere when faced with serious threats to the entire tribe – threats they did not know how to overcome. Some would not move even then, perhaps out of laziness, or because of greed, unwilling to give up the influence or possessions they had accumulated in their existing settlements. Such people usually perished: natural selection tends to favour those willing to act or capable to adapt in times of change. Even so, most people would only move when seriously threatened or in absolute necessity. The rare few who chose to move and explore without any immediate pressure or danger are the true focus of this text, because they were the first ‘scientists’. They were the ones who walked the land, exploring the world. They began to realise that by learning about the world around them and better understanding nature, they could make their lives easier and safer. Gradually, they began to gather knowledge and pass it on to future generations.

Let us now focus on those rare explorers among our distant ancestors. Most of their tribe would likely have believed that there was no need to go anywhere else. They would imagine that other places would be more or less similar to their own. Yet the rare explorers were guided by a different idea: that there could be places much better and safer to live in, with larger, more secure caves and better access to food and water. We can now think of these two beliefs as two opposing scientific hypotheses: the first proposed that no significant differences exist between habitable locations in their region, and the second that some places are significantly better suited for life than their current settlement. While both can be considered valid according to the definition that a hypothesis is ‘the product of an individual’s imagination, based on existing knowledge, that offers a theory that could explain something unknown in nature’, they are mutually exclusive. So, how could our ancestors determine which is closer to the truth?

The early explorers would have to test their hypothesis by conducting an experiment. To do this, they needed to gather new information. This required them to set out on a solitary journey through unfamiliar landscape. They would spend weeks in various locations, examining whether those areas were safer, more hospitable, or offered more abundant food and water. After living for some time in several such places, they might conclude that living conditions elsewhere were, in fact, quite similar indeed. This would make it reasonable for the tribe to stay where they already were. In science, such an outcome is called ‘a negative result’. Yet sometimes, the explorers would return with a clear positive result: they had found a location that was significantly better for settlement, where many of the tribe’s current problems could be more easily addressed, and where they could all live much more safely.

At that point, it was up to a few senior members of the tribe to consider the new information gathered by the explorer and decide what to do. If these influential elders were too rigid, narrow-minded, or attached to their current settlement, they would likely ignore the explorer’s findings. But if they were wise and open-minded, they might follow the explorer and visit the newly discovered location to verify the findings themselves. Once they confirmed the accuracy of the new information, they would accept it as new collective knowledge of the tribe. Then, they would call to action: the members of the tribe should relocate to the significantly better place.

In such a favourable scenario, the explorer’s hypothesis would be accepted based on the new evidence gathered during their solitary expedition. The old belief that all areas in the region were equally habitable would be abandoned. The new information would be verified and validated by respected tribal elders, who had considerable influence over the rest of the group. In this way, new knowledge was created. That knowledge was then applied: a collective decision was made to relocate the entire population. The final step was to implement the plan, whereby the tribe would move from their old settlement to the new one.

This application of new knowledge greatly improved the tribe’s chances of survival. In their new environment, they lived longer and more safely, and their children had better chances of surviving, allowing the tribe to grow and thrive. If there had been no early explorer who imagined a possibility that others had not and who was curious and committed enough to explore the world and gain new knowledge about it, and if the elders had not been wise enough to listen to the explorer, seek confirmation, and accept the result, the tribe would have remained stuck in their unfavourable setting. Eventually, they might have been wiped out by a serious threat, be it a flood, an invasion of wild predators, or famine. That is why these early explorers were the first scientists.

What made them ‘scientists’? First, they asked the question: ‘Could there be a better place for us to live?’ Then, they proposed a new hypothesis: that nature must offer places more suitable for life than those the tribe already knew. After that, they spent time conducting experiments: they tried living in three or four different places, gathering new information about these areas and comparing them to their original habitat. They then acted to expand the tribe’s collective knowledge by sharing their findings with the community. This led to the verification of their insights, followed by the application of knowledge through relocation.

Though this story may seem remarkably simplistic from today’s perspective, we must remember that Earth was once home to several different human species – and all but ours had gone extinct: the Neanderthals, Denisovans, Homo Naledi, the ‘hobbits’ of Southeast Asian islands, and others whose remains are still being unearthed through archaeological excavations. For most of our history, it may have been precisely these kinds of exploratory efforts, followed by the application of new knowledge, that saved our species from the fate that eventually made all the others extinct.

## WHAT MAKES A FIELD OF SCIENCE ‘SUCCESSFUL’?

Although the previous story is set in prehistoric times, all the fundamental principles described in the process of acquiring and applying new knowledge for the benefit of humanity remain valid, even in today’s most advanced areas of research. Every investigation begins with a question, which is usually derived from existing knowledge. The researcher’s imagination draws upon what is already known to offer a new, more accurate interpretation of nature and its phenomena. This new interpretation may or may not be correct, but it must differ from previous beliefs. Next, an experiment is designed with the aim of collecting and analysing new data, based on which two or more competing hypotheses are compared to see which aligns with the observed results most accurately. The experiment highlights the differences in the probabilities of being true between opposing hypotheses. The hypothesis that best matches the measured observations is then accepted, while the others are rejected.

If the accepted hypothesis is based on a completely new idea and an old belief is discarded, this process has created new knowledge. For it to become fully established human knowledge, it must first be validated. This means that other scientists must confirm it before it can be widely accepted. After that, efforts should be made to apply this new knowledge to improve the human condition, compared to the period before the discovery.

Following these principles, we can now ask: what makes one field of science more ‘successful’ than another? In modern science, a field may be considered successful if it attracts large number of researchers. These scientists use their knowledge and imagination to pose many new and interesting questions and develop new hypotheses, some of which would be accepted through experiments, creating large amounts of new knowledge in a relatively short period. The field will experience a high ‘discovery intensity’. In such successful fields, these discoveries are quickly confirmed by other scientists. Once new hypotheses are verified beyond reasonable doubt, the entire scientific community in that field embraces them without resistance. Successful fields also tend to have well-developed pathways and mechanisms for translating and applying new knowledge for the benefit of humanity. Thanks to a successful scientific field, new generations of humans will live safer and better lives. In these fields, scientists responsible for major breakthroughs are duly rewarded and widely recognised for their contributions.

Through understanding these evident principles, the risks that can make a field of science ‘unsuccessful’ also become quite clear. If a field fails to attract a critical mass of educated and imaginative researchers, the pace of creation of new knowledge will remain slow. With fewer motivated scientists, it will take longer for new findings to be independently validated. Once validation occurs, established senior scientists may begin to prioritise their own reputations over the integrity of their field, especially if the new hypotheses challenge their previous work or undermine their authority. In such cases, senior researchers in an unsuccessful field may resist or delay acceptance of new ideas proposed by younger, less established scientists, even when evidence supports the latter. Finally, even when new hypotheses do gain broad acceptance, the field may remain isolated and self-contained, with no well-established mechanisms to apply its discoveries to improve human condition. In the absence of such mechanisms, even the task of applying new knowledge may fall solely on the researchers themselves.

## THE IMPORTANCE OF MEASUREMENT FOR THE ADVANCEMENT OF SCIENCE

Let us return once again to the example of our early ancestor with an exploratory spirit. Clearly, this was a ‘successful’ scientist, whose contribution improved the living conditions and survival prospects of the tribe. But could he have been even more successful, and how? He could have, for example, used measuring tools in his investigations, rather than relying solely on his feet, five senses, and brain. Had he possessed a modern off-road vehicle, he could have visited many more locations in the same amount of time and gained much more information. If he had a pair of binoculars, he could have thoroughly surveyed a range of sites that were otherwise beyond his reach. If, by some miracle, he had access to the satellite imagery we have today, he could have investigated hundreds of potential habitats in greater detail, even gaining a sense of the layout of his entire continent, complete with the positions of rivers, lakes, and impassable mountain ranges.

Supplementary measurement tools can greatly assist us in collecting far more information in the same time span than we could with our five senses only. Had such tools been available to this early explorer, he would have almost certainly identified many more and much better places to live than those he discovered through a few random attempts, mixed with a bit of luck.

In this example, the early explorer had to maintain a degree of faith in his ideas, walk the land on his own, and ‘measure’ each site using his senses to judge how suitable it was for life. Since he could only spend a few days at each location, his conclusions could have easily been flawed. Perhaps he failed to notice that lions or elephants used the same area as a shelter during the rainy or colder seasons. That would have caused major problems for his tribe had it only become known after they had already relocated. The validation by older members of the tribe would have somewhat increased confidence in his observations, since they were more experienced and could have considered such possibilities. Still, there remained a chance that they were all mistaken, simply because they lacked the knowledge needed to make a sound judgment.

What if our prehistoric explorer had access to satellite images, cameras, and powerful computers? He could have monitored thousands of locations at once, across an entire year, and through data analysis generated a vast quantity of valuable information about each site. He could even have defined strict criteria in advance for what makes a habitat ‘good’ for living. Then, he could have focussed his efforts on tracking those specific criteria. If he had a computer at his disposal, he could have integrated and analysed all the collected data much more quickly and presented it clearly to his tribe. In that case, their collective decision to relocate would have been far more justified, compared to one made on very limited information. That decision, crucial for the survival of the entire tribe, would have been made based on a much greater volume and quality of evidence. As a result, they would have had far more confidence in their choice.

From this extremely simple example, we can understand why the advancement of science depends so heavily on researchers’ ability to measure the subjects of their interest and to generate large volumes of data on them. Modern physics is increasingly revealing that the entire universe is, in essence, a vast mathematical system governed by specific laws that can be expressed through mathematical equations. These equations often include certain constant values that never change. If that understanding is close to the truth, then it is not surprising that an effective way to understand all natural phenomena is by measuring events and subjecting them to mathematical and statistical analyses to reach meaningful conclusions. We may come to understand nature best by inventing creative ways to measure it.

Without measurement, the members of our explorer’s tribe might have spent eternity simply discussing and hypothesising about their world; they would still be sitting in their caves, with their lives being no better than before. Human imagination can generate countless theories, stories, legends and specific hypotheses that offer explanations for everything that happens in nature, but without measurement, we cannot truly know anything. In the absence of measurement and experimentation, theories and hypotheses contribute no more to human progress than the old Native American legend about the origin of the Moon.

## OUR SENSES AND OTHER MEASURING TOOLS

To test their theories and hypotheses through impartial data collection, ideally through measurement within well-designed experiments, scientists will often require reliable tools. There have been instances in which scientists developed and confirmed hypotheses that stood the test of time for centuries, appearing to explain the observed phenomena remarkably well. However, over even longer periods, small discrepancies from the prevailing hypothesis would begin to surface, or rare exceptions to accepted rules would be carefully documented. These exceptions or anomalies are often the most interesting to researchers. They can signal that the currently accepted theories and hypotheses remain only ‘partial’, and there are some more fundamental, deeper, and wider truths that still need to be understood. The current theories may explain most observations under certain conditions, but they may start to fail in unusual circumstances. In many cases, people have been able to replace these early, partial theories with more generalised and fundamental laws once they gained the ability to measure more precisely, thereby accessing far greater volumes of information.

Our five primary senses evolved to help us survive on the surface of the Earth. Although our planet exists within an unimaginably vast universe, we spend our lives confined to its relatively thin, hardened crust, embraced by an atmosphere that provides oxygen, shielded by a magnetic field that protects us from cosmic radiation, with molten lava beneath us and a cold, airless, oxygen-starved space-time continuum surrounding our only habitat. Without additional tools, we are simply not equipped to understand much about the internal structure of the Earth, let alone the Sun, Moon, or other celestial bodies, or the vast cosmos in which we drift. Our senses evolved to navigate our limited habitat, not to enable us to study and comprehend the universe. The visible light spectrum that our eyes can detect is only a narrow sliver of all electromagnetic waves, and we cannot see many of them.

Other living beings on Earth have developed entirely different sensory capabilities. Bats possess echolocation – the ability to orient themselves by interpreting reflected sound waves, much like sonar – allowing them to navigate in total darkness [2]. Perhaps even more impressively, several species of marine animals can sense changes in nearby electric fields, a faculty called electroreception [3]. Birds and bees can orient their flight using magnetoreception [4], sensing the Earth’s magnetic field. Cuttlefish can perceive polarised light and use it to navigate on cloudy days [5]. Plants have sensory mechanisms that detect vibrations, light, water, scents, and even specific chemicals in their surroundings [6]. Clearly, various species have evolved a wide array of sensory systems to gather information from the environment and survive. It is thus worth remembering that much less than 1% of all species that have ever lived on Earth are still alive today, as the rest have already gone extinct [7].

If we accept that humans evolved in their current form primarily to survive and reproduce on Earth’s surface, and not to study the laws of the universe and nature, then we cannot know what lies beyond the limits of our senses, though such phenomena could – and should – be studied. If we lacked hearing, we could still survive, but we would likely struggle to even imagine what sounds are. Therefore, it made sense for humans to begin investigations of nature by observing and measuring the phenomena that their senses allowed them to perceive. However, those studies likely represented only a miniscule fraction of the information that the universe has to offer. That is why, in the next step, humans needed to develop tools and technologies that allowed them to extend the potential of their senses to observe and measure natural phenomena. Even more excitingly, they could try to create entirely new, artificial ‘senses’ by building measurement devices to study phenomena outside of the spectrum of human sensory perception. For example, the development of the microscope was an extension of our sense of vision [8], allowing us insights into tiny worlds and giving rise to fields like microbiology and histology, which could never have developed through unaided human eyesight. The invention of the telescope, which also enhances our vision, enabled the development of astronomy [9]. From radar, sonar, spectrometers, and atomic clocks to polymerase chain reactions, the Large Hadron Collider (LHC) and the Laser Interferometer Gravitational-wave Observatory (LIGO), these are all incredibly useful tools and methods that humans now use to observe nature and perform measurements that their limited senses could not do on their own. Many of these instruments are enhancements of our existing senses, but some are entirely novel sensory extensions.

A key stage in the development of any science is the ability to find ways to objectively measure phenomena of interest. Even the most complex or abstract problems, such as ‘quality of life’ in the social sciences, can be explored and become a serious subject of scientific inquiry once tools for their measurement are introduced. In this particular case, a survey instrument called the Short Form-36 (SF-36), which is a simple set of 36 carefully designed and validated questions, enables the measurement of eight essential dimensions of an individual’s everyday functioning, from physical to psychological to social [10]. It has made possible a whole body of research on quality of life in human communities.

Another powerful example of the importance of measurement in scientific progress is the history of the microscope. Richard Zsigmondy developed the ultramicroscope, which enabled the study of structures smaller than the wavelength of light and earned him the Nobel Prize in Chemistry in 1925. Then, Frits Zernike invented the phase-contrast microscope which allowed scientists to examine colourless and transparent biological materials, and thus won the Nobel Prize in Physics in 1953. Ernst Ruska developed the electron microscope, dramatically improving image resolution in micro-worlds and expanding their study, for which he was awarded the Nobel Prize in Physics in 1986. Later, Gerd Binnig and Heinrich Rohrer invented the scanning tunnelling microscope, enabling three-dimensional imaging of objects down to the atomic level. This earned them the 1986 Nobel Prize in Physics, which they shared with Ruska.

Even in recent times, the refinement of the microscope remains a gateway to Nobel recognition. In 2014, Eric Betzig, Stefan W. Hell, and William E. Moerner won the Nobel Prize in Chemistry for overcoming the presumed 0.2-µm resolution limit of optical microscopes. They developed advanced microscopy techniques using molecular fluorescence, allowing scientists to observe interactions between individual molecules inside living cells. This made it possible, for instance, to watch protein clusters associated with disease form or to track different stages of cell division. In short, these are five separate Nobel Prizes, all awarded for advances in the same area: the enhancement of human vision, enabling us to explore worlds far smaller than ourselves [11,12].

## THE RISE OF COMPUTERS, BIG DATA, AND HYPOTHESIS-FREE SCIENCE

For centuries, science advanced through a sequence of intellectual steps that had become almost ritualistic: observation was followed by a hypothesis, leading to an experiment, analysis, conclusion, validation and application. This cycle, familiar to any scientist, served as the bedrock of how new knowledge was discovered, tested, and applied. While elegant, this process was usually slow and deeply reliant on the imagination and skill of individual researchers. Yet in the 21st century, this process has undergone a remarkable transformation. Thanks to the rise of computers, the proliferation of massive datasets, and the development of hypothesis-free approaches to research, the traditional sequence of steps leading to scientific discoveries was disrupted – sometimes subtly, but sometimes quite dramatically.

At the heart of this shift lies a fundamental change in how science begins. In classical science, progress typically started with a hypothesis developed by an individual scientist: a carefully framed idea based on prior knowledge, theory, or intuition. This hypothesis was then tested through controlled experiments using targeted data. Today, in many fields, from genomics and astronomy to social science and economics, the order has been reversed. Data now comes first, followed by the analyses and the results. The correct answers can then be seen before the related research questions were even posed.

This new model is often described as ‘hypothesis-free science’ or ‘data-driven discovery’. Rather than proposing a single idea to test, researchers begin by gathering vast amounts of information, often through automated or high-throughput systems. These might include genome sequencers producing precise reads of billions of DNA base pairs, satellites scanning every corner of the Earth, or digital sensors capturing every click, purchase, and movement of billions of humans. Once the data are collected, powerful algorithms and statistical models are used to scan for patterns, correlations, trends, and anomalies. Computers can, in effect, test millions of potential hypotheses in parallel – rapidly, impartially, systematically, and exhaustively.

A seminal example of this approach can be found in the field of genetics. For decades, geneticists worked on a ‘candidate gene’ model. They would identify specific genes that might plausibly influence a trait, based on biological reasoning. They would then test genetic variants in those genes for association with the trait of interest. But the advent of the Human Genome Project and the subsequent rise of genome-wide association studies (GWAS) turned this process on its head. Instead of focussing on a few genes, researchers now analyse millions of single nucleotide polymorphisms (SNPs) across the entire genome, with no preconceived notions of which regions might be important. These studies can reveal genetic associations with diseases, behaviours, or traits that no biologist would have thought to investigate [13].

Crucially, this new approach has allowed science to move beyond the limitations of human intuition. While the traditional scientific method relies heavily on human cognitive capacity, such as pattern recognition, analogical thinking, and creativity, it is also constrained by our cognitive blind spots. We can only formulate hypotheses about what we already understand, or at least about what we can imagine. Hypothesis-free science, powered by big data and computing, opens the door to discoveries that are truly unexpected, revealing relationships that lie outside the possibly quite narrow field of human anticipation.

The implications of this new era for science are vast. In medicine, this means identifying biomarkers for diseases long before symptoms appear. In climatology, it means using satellite imagery and climate models to detect tipping points in Earth’s systems. In economics and social science, it means analysing real-time data from millions of individuals to better understand behaviour, inequality, or the spread of misinformation. In socio-linguistics and other humanities, analyses of millions of books through history can reveal the rise and fall of ideas, concepts, or even individual’s fame, all of which can now be measured. In fundamental physics, it means combing through terabytes of particle collision data to find evidence of particles or forces not predicted by existing theories.

Yet this revolution is not without its challenges. The first one is statistical: when testing millions of hypotheses, many associations will appear significant by chance alone. This raises the risk of ‘false positive’ results in science, *i.e.* confirmed results that appear real, but are not reproducible. Traditional science guards against this through careful experimental design, replication, and stringent statistical tests. In hypothesis-free science, the challenge is managing and interpreting statistical significance at scale. Techniques like Bonferroni correction, false discovery rate control, and cross-validation have become essential tools in the data scientist’s arsenal, but they are not perfect [14].

The second challenge is interpretability. Discovering a correlation in data is not the same as understanding causation. A GWAS study might identify a variant associated with disease risk, but what does it mean? How does that variant affect cellular function, protein expression, or immune response? Data may point us to where something interesting is happening. However, explaining those findings often requires returning to traditional scientific approaches: experiments, models, and mechanisms. In this way, hypothesis-free science is not a replacement for classical science, but a powerful new front end. It is a generator of clues and surprising new truths that can then be followed up with deeper, hypothesis-driven research.

The third challenge is cultural. The rise of big data science has altered the skills that researchers need. Programming, machine learning, database management, and statistical inference are now central to modern scientific endeavour, even in fields that were once firmly rooted in qualitative or descriptive methods. While the lone scientist with a notebook and a microscope is still a vital part of the ecosystem, important new discoveries increasingly emerge from interdisciplinary teams that managed to integrate all important elements that lead to discovery – technologies and skilled humans alike. Modern teams that are highly competitive in science assemble data engineers, statisticians, domain experts, ethicists, and communicators, working together across vast networks. The boundaries between disciplines are blurring, multi- and trans-disciplinarity is a new strength, and success now often depends on good collaboration, not just individual insights.

There are also philosophical implications. What happens when we find the correct answers before we even asked the related research questions? What does it mean for science to become less about theory and more about detection? Will hypothesis-free science turn into ‘fishing expeditions looking for correlations’ and encourage a rather incomplete form of inquiry, in which explanations will start to lag far behind key discoveries, rather than co-occur with them, as they did historically. Still, data-driven science has already revealed regularities so robust that they have reshaped entire fields, despite lacking complete theoretical explanations.

Importantly, the impact of this new approach is not confined to elite research institutions. Thanks to open-access repositories, cloud computing, and public datasets, hypothesis-free science has become democratised. A young researcher in low- or middle-income country with a laptop and internet connection can now analyse the most expensive and informative global health datasets, satellite imagery, or genomic data alongside colleagues from leading universities in high-income countries. This accessibility is helping us address global disparities in scientific opportunity. It could lead to accelerated innovation due to a more diverse spectrum of ideas [15].

Data privacy, consent, algorithmic bias, and ethical oversight are all potential concerns in this new landscape. Large datasets often contain sensitive personal information, and algorithms trained on biased data can perpetuate or even worsen existing social inequalities. Hypothesis-free science must be accompanied by rigorous ethical frameworks and transparent governance to prevent the creation of tools that are powerful, but unaccountable.

In sum, the rise of computers, big data, and hypothesis-free science has taken the humanity into a new era of discovery. It does not replace the traditional scientific method, but rather extends its reach, allowing us to scan the unknown with unprecedented speed and breadth. We can now let our data speak first, and then curiously follow the leads that we obtain. In the long history of scientific discovery, this may eventually prove to be of the most important transitions.

## THE RISE OF ARTIFICIAL INTELLIGENCE AND THE EMERGENCE OF NON-HUMAN SCIENTISTS

Each major leap in the history of science has followed from the success of a powerful new tool for observation and measurement. Telescopes allowed us to see the stars, microscopes revealed the microbes, and computers transformed how we store, process, and analyse data. Moving further into the 21st century, a new tool has entered the stage, and it might prove to be the most transformative of all: artificial intelligence (AI).

AI refers to the development of systems that can perform tasks typically requiring human intelligence – such as learning, reasoning, pattern recognition, natural language understanding, and decision-making. Over the past decade, AI has completed a transition from a theoretical pursuit into a practical, fast-evolving, and increasingly useful tool in science. Yet unlike earlier tools, which extended our senses or computational capabilities, AI is beginning to extend our cognitive abilities. It can generate research ideas, propose models, notice patterns that are invisible to us, and even write research papers [16]. It may become the first non-human scientist, surpassing humans in many of their abilities as researchers. This profound change may have the potential to fundamentally reshape science as we know it.

AI is already making science more successful by accelerating every stage of the research process. Historically, in drug discovery, developing a new drug required many years of trial-and-error experimentation, with high costs and frequent failures. Today, AI can analyse the molecular structures of millions of compounds, predict their likely interactions with target proteins, and suggest novel candidates for laboratory testing [17]. Google's DeepMind developed an AI system called AlphaFold that revolutionised our understanding of protein folding, which was a major challenge in biology. For decades, biologists struggled to predict the three-dimensional structure of proteins based on their amino acid sequences. AlphaFold solved this problem with unprecedented accuracy, providing a foundation for breakthroughs in medicine, agriculture, and environmental science. This is a striking example of how AI does not just accelerate science, but can also solve problems long considered intractable to human brain [18,19].

In fields like materials science, AI is enabling the design of new materials with desired properties. In astronomy, telescopes and space probes now generate more data than any team of human astronomers could possibly analyse. AI algorithms are being trained to scan this data for signs of missed exoplanets, gravitational waves, or unusual galactic structures. In particle physics, too, AI is helping sift through the massive outputs of experiments like those at CERN to spot rare events that may indicate new particles or forces [20].

However, AI's transformative potential lies not only in data analysis, but in hypothesis generation itself. In the history of science, first came philosophers, forming hypotheses about the world. Then came experimenters, who assigned likelihoods to all the hypotheses that humans ever proposed, thus generating what we refer to as ‘knowledge’. Traditionally, forming a good hypothesis based on all previous knowledge has been one of science’s most human and creative acts. Now, humans have generated so much knowledge in so many fields of science that no single human can grasp even a tiny fraction of it when proposing new hypotheses. Howeverlarge language models (LLMs) have been trained on all written knowledge produced by humans. It is intriguing to predict that they could now become ‘philosophers 2.0’, using the entire human knowledge to generate plausible scientific hypotheses. In doing this, they can synthesise enormous volumes of literature, identify gaps or inconsistencies, and propose new lines of inquiry. In some research institutions, LLMs are already being used as collaborative partners, scanning vast biomedical databases, designing experiments, and even suggesting interpretations of results – leading to them eventually becoming ‘scientists 2.0’.

The ‘science 2.0’ could become a phase of collaboration between humans and AI-based machines, where humans would still be expected to bring in context, ethics, and domain intuition, while AI would provide enhanced memory and scale of scientific inquiry, and computational reasoning. ‘Science 2.0’ could be based on this hybrid intelligence, that should be capable of exploring the scientific unknowns in ways that no single human mind ever could.

All these developments also raise some critical questions. One is the issue of explainability, because at this point in time AI systems – especially models based on so-called ‘deep learning’ – often operate as ‘black boxes’. They seem to produce results that are accurate, sometimes even surprisingly so, but not always understandable. In science, where explanation and transparency are foundational, this presents a dilemma about whether we should ever trust a conclusion that we, humans, cannot understand or explain? This is why research into developing tools to enable AI interpretability is evolving fast, to ensure that scientific insights remain not only useful, but also fully meaningful to humanity as users of this new knowledge.

Another challenge is potential bias. The quality of AI systems depends on the data they are trained on. If the training data contains historical biases, omissions, or errors, the AI can reproduce those problems or even amplify them. In medicine, for example, training diagnostic models only on data from Western populations may lead to poor performance when applied elsewhere in the world. Therefore, the global scientific community must work to ensure that AI systems are trained on diverse, representative, and high-quality datasets.

One of the entirely novel challenges for editors of scientific journals is that the use of AI to improve science will force us to rethink issues of authorship, credit, and accountability [16]. If a hypothesis is generated by an AI, who deserves credit, then? If a paper is co-written with a language model, who is its true author? What if AI wrote a grant proposal? Scientific norms around originality, contribution, and peer review will need to be re-examined in light of these new tools. Some journals have begun to issue guidelines for the use of AI in manuscript preparation or data analysis, signalling that the integration of AI into science must not only add power, but also be principled [15].

Although these challenges will need to be carefully addressed, the trend is rather clear. The use of AI will make science more successful. New results and new knowledge will be generated much faster, they will be based on far more information than before, and that information will be much better integrated and connected. AI will enable us to study complexities of life and nature at scales once thought impossible, uncovering networks and relationships across many scientific disciplines. It will help researchers identify previously overlooked variables and their interactions, simulate complex systems, and personalise medical interventions. One of the most exciting opportunities that lies ahead is AI’s potential to unify science across its disciplines, which no human could at this day and age. Historically, much of human scientific progress has been divided into, for example, physics, chemistry, biology, materials science, computer science, and many other fields. Yet the nature that surrounds us is, in fact, not divided in this way – it contains all of those fields at once, interacting with each other. AI, with its capacity to find patterns across highly heterogeneous data, may help humans to bridge these divides. Already, AI is being used to map relationships between seemingly unrelated datasets, such as combining climate data with epidemiological data to predict future disease outbreaks under global warming scenarios [21].

A key new area of progress where AI can make a substantial impact is scientific communication and mass-education. Namely, LLMs can translate highly complex findings into readable summaries that are approachable and understandable to almost anyone. They can generate educational materials and answer complex questions on science in plain language. This should enable not only better understanding of science by the general public, but also better communication between scientists across different fields and quick entry into specific fields for researchers with novel ideas [22]. This has a substantial potential to democratise science, making it accessible to non-specialists, students, and policymakers alike, and empowering public understanding and participation.

Looking ahead, the continued development of AI could also make science more proactive than reactive. Instead of waiting for scientific problems to emerge, AI-enhanced systems can scan the horizon for weak signals, simulate cascading risks, and suggest pre-emptive strategies against any looming collapses that threaten the humanity. In this sense, it may not only make science more successful than ever before, but also more humane: focussed not only on generating new knowledge for its own sake, but also on applied and meaningful foresight and proactive resilience to benefit humanity, based on information of the greatest value to human beings [23].

In the history of science’s successful generation of human knowledge, AI becomes a tool that not just improves human senses like a microscope or a telescope, or provides additional ones like LHC or LIGO, or extends human memory and computational capacity like computers do, but that expands human reasoning itself. AI tools are capable of generating hypotheses and offering study designs that no human has ever thought of, revealing patterns that we cannot see and pointing us toward answers that would have stayed beyond our reach without the assistance from AI. Therefore, the rise of AI does not signal the end of the scientific method, but rather its evolution. In this new phase, science will remain a deeply human interest, arising from curiosity and wonder and guided by principles of ethics. But in the next steps, humans will be joined by a new kind of intelligence, trained on the collective knowledge that the humanity managed to generate so far, and able to assist us to reach further than we thought possible, to make science even more successful based on all the key criteria proposed in this paper.

## APPLYING NEW KNOWLEDGE TO IMPROVE HUMAN LIVING CONDITIONS

Even if we agree that instruments and tools that can measure and analyse collected information are critically important for scientific progress, this still does not mean that the very act of measuring something in nature necessarily qualifies as ‘doing science’. Imagine, for example, that we own a house with a lovely garden and two trees. In springtime, our brain’s sense of ‘perception of ideas’ [24] might suddenly favour our idea to count the number of leaves on each of the two trees. In doing so, we would generate new information about which tree has more leaves. But is that scientific work?

This is not an easy question to answer. There might be a valid scientific reason for counting tree leaves. For instance, someone might have a hypothesis that urban air pollution negatively affects trees, and one way to test that hypothesis could be by counting the number of leaves on each tree over several years. Therefore, if there is a clear scientific question that motivates the measurement and a vision for how the newly generated information might be applied, the act of measuring gains purpose and becomes a part of scientific process.

In contrast, measuring natural phenomena without a clear hypothesis or application becomes self-contained and likely remains isolated from the rest of science – unused and forgotten. That said, it is also possible that such data may someday prove valuable within a future study [25,26]. The history of science has shown many times already that it is remarkably difficult to predict in advance which measurements and of which phenomena will genuinely advance science or lead to meaningful progress for humanity. There are many examples where major breakthroughs came from what initially appeared to be random or obscure investigation, one that most scientists at the time overlooked, but which later turned out to be immensely significant thanks to precise measurements.

In conclusion, we should learn to recognise what makes science ‘successful’ and acknowledge its accomplishments. It is truly astonishing how much science and technology have transformed human life over the past few centuries. Although we did not evolve in this biological form to conduct fundamental scientific research, it is precisely science that has allowed us to shield ourselves from the influence of most other life forms and most natural forces, to extend our lifespan, increase our average height and intelligence, and to meet all of our basic needs with far greater ease than our ancestors ever could.

We have learned how to build warm and safe shelters regardless of the weather outside, to light them up regardless of time of day, and to construct settlements in which dangerous animals no longer pose a threat. We have brought clean drinking water into our homes, installed sanitation systems, and made food, education, and healthcare widely accessible. Technology has enabled us to move across the Earth’s surface faster and more easily, through cars, trains, ships, and airplanes. It has enabled us to communicate and to access all human knowledge and information through the development of computers and the internet. Now we have a new companion – AI – to assist us in planning the path ahead. Thanks to the achievements of scientists and innovators who have always been just a tiny fraction – less than 1% – of the total human population, the majority of us are living much safer, healthier, more educated, and longer lives than any generation since the first humans appeared on our planet.
